# Aortic Valve Replacement With Mechanical Valves vs Perimount Bioprostheses in 50- to 69-Year-Old Patients

**DOI:** 10.1016/j.jacadv.2023.100359

**Published:** 2023-06-07

**Authors:** Ruixin Lu, Michael Dismorr, Natalie Glaser, Ulrik Sartipy

**Affiliations:** a Department of Molecular Medicine and Surgery, Karolinska Institutet, Stockholm, Sweden; b Department of Cardiothoracic Surgery, Karolinska University Hospital, Stockholm, Sweden; c Department of Cardiology, Stockholm South General Hospital, Stockholm, Sweden

**Keywords:** aortic valve replacement, bioprosthetic valves, cardiac surgery, mechanical heart valves, middle-aged, epidemiology

## Abstract

**Background:**

Evidence is mixed regarding the most appropriate type of valve prosthesis for surgical aortic valve replacement (AVR) in patients 50 to 69 years. American and European guidelines differ.

**Objectives:**

The purpose of this study was to determine the long-term all-cause mortality and complication rates after AVR in patients aged 50 to 69 years according to implantation of a Perimount bioprosthetic valve or a mechanical valve.

**Methods:**

In this nationwide observational cohort study, all patients aged 50 to 69 years who underwent primary surgical AVR in Sweden 2003 to 2018 using a Perimount bioprosthesis or mechanical valve were identified from the SWEDEHEART register. Primary outcome; all-cause mortality, secondary outcomes; major bleeding, aortic valve reintervention, heart failure hospitalization, and stroke. National health-data registers were used to ascertain outcomes. Regression standardization addressed confounding.

**Results:**

A total of 6,907 patients aged 50 to 69 years were included (Perimount group, n = 3,831 and mechanical valve group, n = 3,076) and 74% were men. The use of bioprostheses increased during the study period. At 15 years of follow-up, the estimated cumulative incidence of all-cause mortality was 37% (95% CI: 35%-40%) vs 45% (95% CI: 42%-48%) in the mechanical and Perimount groups, respectively (survival difference −7.9% [95% CI: −11% to −4.6%]). Patients with mechanical valves had a lower risk of aortic valve reintervention but a higher risk for bleeding. Survival difference at 15 years in ages 50 to 59 years was −15% (95% CI: −8.4% to −21%).

**Conclusions:**

In patients aged 50 to 69 years who underwent surgical AVR, survival was better in those who received mechanical compared to Perimount bioprosthetic valves. While valve choice should be guided by individual patient factors and patient preference rather than by chronological age, the substantial survival advantage observed in patients with mechanical valves in ages 50 to 59 years must be recognized.

Untreated severe aortic valve stenosis has an annual mortality rate of up to 25% and a mean survival of 2 to 3 years.^1^^,^^2^ Aortic valve replacement (AVR) is the only definitive treatment and can be performed by open surgery or transcatheter aortic valve replacement (TAVR). Although AVR is life-saving, the loss of life expectancy following AVR is approximately 3 to 4 years in patients aged 50 to 69 years when compared with that in the general population.^3^ It has been debated whether a mechanical or biological aortic valve prosthesis should be used in patients aged 50 to 69 years,^4^ and the results of previous studies have been contradictory,^5^, ^6^, ^7^ leading to different recommendations in the American and European guidelines.^8^^,^^9^ Comparative long-term performance studies of aortic bioprostheses have shown wide variation among the various bioprosthetic valve models.^10^^,^^11^ In a previous study using data from the SWEDEHEART (Swedish Web-system for Enhancement and Development of Evidence-based care in Heart disease Evaluated According to Recommended Therapies) register,^12^^,^^13^ we observed that survival was better in patients aged 50 to 69 years who underwent surgical AVR using a mechanical valve than in those who received a bioprosthetic valve.^6^ In a subsequent SWEDEHEART study, we found that long-term performance and survival was better when a Carpentier-Edwards PERIMOUNT valve (Edwards Lifesciences Corp), was placed than when other bioprosthetic valves were used.^11^ In view of our results,^6^^,^^11^ we thought it worthwhile to investigate if the best bioprosthesis could outperform a mechanical valve in patients aged 50 to 69 years who require AVR. Therefore, we performed this observational cohort study to determine the long-term all-cause mortality, bleeding, heart failure hospitalization, aortic valve reintervention, and stroke rates after AVR in patients aged 50 to 69 years according to whether a Perimount bioprosthetic valve or a mechanical valve was used.

## Methods

### Study design

The protocol for this observational, nationwide, population-based cohort study was approved by the Swedish Ethical Review Authority. The requirement for informed consent was waived because the data were deidentified. Study reporting followed the STrengthening the Reporting of OBservational studies in Epidemiology and REporting of studies Conducted using Observational Routinely collected health Data guidelines.^14^^,^^15^

### Study population and data sources

All patients aged 50 to 69 years in Sweden who underwent surgical AVR with a Perimount bioprosthesis or mechanical valve with or without concomitant coronary artery bypass grafting or ascending aortic surgery between January 1, 2003, and December 31, 2018 were included in the study. Patients were excluded if they met any of the following criteria: age younger than 50 years or older than 69 years, previous cardiac surgery, previous TAVR, concomitant surgery on another valve, use of deep hypothermia and circulatory arrest, undetermined type of prosthesis, or use of a bioprosthetic valve other than a Perimount. The study population was identified from the Swedish Cardiac Surgery register, which is a part of the SWEDEHEART registry.^12^ The Swedish Cardiac Surgery Register records all patients who have undergone cardiac surgery in Sweden since 1992 and contains preoperative, perioperative, and postoperative data, including survival status. The Swedish Cardiac Surgery Register has been shown to have high reliability and validity.^13^ The Swedish National Patient Register was used to obtain baseline characteristics, including comorbidities and bleeding, hospitalization for heart failure, aortic valve reintervention, and stroke outcomes. The heart failure and stroke diagnoses in the National Patient Register have also been shown to have high reliability and high validity.^16^^,^^17^ Socioeconomic background characteristics were obtained from the longitudinal integrated database for health insurance and the labor market studies, maintained by Statistics Sweden.^18^ The Swedish personal identity number made it possible to cross-link data at the individual level.^19^ These national registers have been described elsewhere.^6^

### Exposure and outcomes

The patients were categorized as having either a mechanical valve or a Perimount bioprosthesis. The Perimount bioprostheses included the 2900, Magna 3000, and Magna Ease 3300 models. The valve models and their frequency of use are listed in Supplemental Table 1. The primary outcome was all-cause mortality, as obtained from the Swedish Population Register.^20^ Secondary outcomes were the cumulative incidence of bleeding, aortic valve reintervention (defined as a subsequent surgical AVR or TAVR), heart failure hospitalization, and stroke. The International Classification of Diseases-10 codes used to ascertain each outcome were obtained from the National Patient Register and are presented in Supplemental Table 2.

### Statistical analysis

Categorical variables are presented as the frequency (percentage) and continuous variables as the mean ± SD. Time to event was defined as the number of days from the date of surgery until the date of the event or the end of follow-up. The end of follow-up for death was March 17, 2020 and that for the secondary outcomes was December 31, 2018. The Kaplan-Meier method was used to calculate the crude survival. The Aalen-Johansen estimator was used to estimate the crude cumulative incidence of bleeding, heart failure hospitalization, reintervention, and stroke while accounting for the competing risk of death. A Poisson model was used to obtain age- and sex-adjusted incidence rates. To account for differences at baseline, the standardized cumulative survival and differences in survival were estimated using flexible parametric regression standardization. The resulting survival curve estimates the population outcome if the entire population would have received each respective type of valve. This method adjusts for the population distribution of covariates.^21^^,^^22^ Model selection was performed using clinical subject matter knowledge and a backward selection strategy informed by the Akaikes information criterion. Details of the final models are available in the Supplemental Appendix. Flexible hazard-based regression standardization was used to estimate the cumulative incidence and differences in bleeding, reintervention, heart failure hospitalization, and stroke rates as described by Kipourou et al.^23^ The resulting cumulative incidence curve estimates the population outcome, had the entire population received one or the other type of valve. This method adjusts for the population distribution of covariates while accounting for the competing risk of death. The Classification and Regression Tree estimation and imputation approach was used to handle missing data.^24^ As a sensitivity analysis, the main analyses were repeated using an inverse probability of treatment weighting (IPTW) approach. Stabilized weights were obtained using generalized boosted regression models.^25^ We also performed subgroup analyses according to whether patients were aged 50 to 59 years or 60 to 69 years and in the subset of patients who underwent isolated AVR. All statistical analyses and data management were performed using the R programming language, version 4.2.0 (R Foundation for Statistical Computing), and included the use of the “mexhaz,” “rstpm2,” and “WeightIt” packages.^21^^,^^26^^,^^27^

## Results

In total, 6,907 patients aged 50 to 69 years who underwent surgical AVR in Sweden during the study period and fulfilled the inclusion criteria were identified (Perimount group, n = 3,831; mechanical valve group, n = 3,076). The numbers of mechanical and Perimount AVRs carried out per year in Sweden during the study period are shown in Supplemental Figure 1. The number of implants per year according to subtype of Perimount bioprosthesis are shown in Supplemental Figure 2. The mean age was 61.9 years (standard deviation 5.2) and 74% of the study population were men. There were small but potentially important baseline differences between the groups; eg, the mean age was 64 years in the Perimount group and 59 years in the mechanical valve group. The following variables had missing data: body mass index (4.9%), estimated glomerular filtration rate (1.9%), education level (0.9%), left ventricular ejection fraction (0.9%), emergent surgery (0.9%), valve size (0.7%), and household income (<0.01%). The baseline characteristics are presented in Table 1.

**Table 1:** Baseline Characteristics in Patients Aged 50 to 69 Who Underwent Surgical Aortic Valve Replacement With a Perimount or Mechanical Valve Between 2003 and 2018 in Sweden

	Overall (N = 6,907)	Perimount (n = 3,831, 55%)	Mechanical (n = 3,076, 45%)
Age, y	61.9 ± 5.2	64.0 ± 4.3	59.3 ± 5.0
Male	5,086 (73.6)	2,791 (72.9)	2,295 (74.6)
Married	4,165 (60.3)	2,324 (60.7)	1,841 (59.9)
Education			
<10 y	2,165 (31.5)	1,225 (32.1)	940 (30.8)
10-12 y	3,053 (44.5)	1,647 (43.2)	1,406 (46.0)
>12 y	1,649 (24.0)	939 (24.6)	710 (23.2)
Non-Nordic birth region	518 (7.5)	252 (6.6)	266 (8.6)
Household income			
Q1 (lowest)	1,055 (15.3)	616 (16.1)	439 (14.3)
Q2	1,359 (19.7)	731 (19.1)	628 (20.4)
Q3	1,814 (26.3)	948 (24.8)	866 (28.2)
Q4 (highest)	2,678 (38.8)	1,535 (40.1)	1,143 (37.2)
Body mass index, kg/m^2^			
<18.5	50 (0.8)	33 (0.9)	17 (0.6)
18.5-24.9	1,873 (28.9)	1,054 (28.8)	819 (29.0)
25-29.9	2,781 (42.8)	1,567 (42.8)	1,214 (42.9)
≥30	1,787 (27.5)	1,008 (27.5)	779 (27.5)
Diabetes mellitus	1,279 (18.5)	809 (21.1)	470 (15.3)
Prior atrial fibrillation	909 (13.2)	499 (13.0)	410 (13.3)
Hypertension	3,292 (47.7)	2,046 (53.4)	1,246 (40.5)
Hyperlipidemia	1,470 (21.3)	874 (22.8)	596 (19.4)
Prior stroke	573 (8.3)	373 (9.7)	200 (6.5)
Peripheral vascular disease	1,056 (15.3)	556 (14.5)	500 (16.3)
COPD	626 (9.1)	401 (10.5)	225 (7.3)
Prior myocardial infarction	815 (11.8)	477 (12.5)	338 (11.0)
Prior PCI	477 (6.9)	311 (8.1)	166 (5.4)
Pacemaker/ICD	134 (1.9)	79 (2.1)	55 (1.8)
Prior major bleeding event	434 (6.3)	314 (8.2)	120 (3.9)
Alcohol dependence	302 (4.4)	201 (5.2)	101 (3.3)
Hepatic disease	122 (1.8)	90 (2.3)	32 (1.0)
History of cancer	628 (9.1)	417 (10.9)	211 (6.9)
eGFR, mL/min/1.73 m^2^			
<30	156 (2.3)	99 (2.6)	57 (1.9)
30-44	153 (2.3)	112 (3.0)	41 (1.4)
45-59	590 (8.7)	390 (10.4)	200 (6.7)
≥60	5,868 (86.7)	3,159 (84.0)	2,709 (90.1)
Prior heart failure	1,242 (18.0)	714 (18.6)	528 (17.2)
Prior endocarditis	475 (6.9)	269 (7.0)	206 (6.7)
LVEF			
<30%	398 (5.8)	238 (6.3)	160 (5.2)
30%-50%	1,357 (19.8)	744 (19.6)	613 (20.1)
>50%	5,098 (74.4)	2,822 (74.2)	2,276 (74.6)
Emergent operation	136 (2.0)	79 (2.1)	57 (1.9)
Isolated AVR	4,130 (59.8)	2,331 (60.8)	1,799 (58.5)
Concomitant CABG	1,641 (23.8)	1,003 (26.2)	638 (20.7)
Ascending aortic surgery	1,337 (19.4)	589 (15.4)	748 (24.3)
Valve size, mm			
18-21	1,500 (21.9)	841 (22.0)	659 (21.8)
22-23	2,568 (37.5)	1,447 (37.9)	1,121 (37.0)
24-29	2,778 (40.6)	1,531 (40.1)	1,247 (41.2)
Period of surgery, y			
2003-2008	2,188 (31.7)	777 (20.3)	1,411 (45.9)
2009-2013	2,166 (31.4)	1,310 (34.2)	856 (27.8)
2014-2018	2,553 (37.0)	1,744 (45.5)	809 (26.3)

Values are mean ± SD or n (%).

AVR = aortic valve replacement; CABG = coronary artery bypass graft; COPD = chronic obstructive pulmonary disease; eGFR = estimated glomerular filtration rate; ICD = implantable cardioverter-defibrillator; LVEF = left ventricular ejection fraction; PCI = percutaneous coronary intervention; Q = quartile.

### Clinical outcomes

The crude and age- and sex-adjusted incidence rates for all outcomes are shown in Supplemental Table 3. The regression standardized cumulative incidence of all outcomes at 15 years is shown in Table 2 and at 5 and 10 years in Supplemental Tables 4 and 5.

**Table 2:** Regression Standardized Cumulative Incidence at 15 Years of All-cause Mortality, Bleeding Events, Aortic Valve Reintervention, Heart Failure Hospitalization, and Stroke After Surgical Aortic Valve Replacement With a Perimount or Mechanical Valve in Patients Aged 50-69 Years, 50-59 years and 60-69 Years

	Cumulative Incidence (95% CI)		Cumulative Incidence Difference (95% CI)
	Perimount	Mechanical	Mechanical vs Perimount
All-cause mortality			
Age 50-69 y	45 (42-48)	37 (35-40)	−7.9 (−11 to −4.6)
Age 50-59 y	41 (35-47)	27 (24-30)	−15 (−21 to −8.4)
Age 60-69 y	47 (44-50)	42 (39-45)	−4.8 (−8.6 to −1.0)
Bleeding			
Age 50-69 y	9.7 (8.1-12)	16 (14-19)	6.6 (4.1-9.2)
Age 50-59 y	7.8 (5.0-12)	15 (12-18)	7.1 (3.0-11)
Age 60-69 y	11 (8.9-13)	17 (14-20)	6.4 (3.4-9.5)
Aortic valve reintervention			
Age 50-69 y	13 (10-17)	5.9 (4.4-7.8)	−7.3 (−10 to −4.5)
Age 50-59 y	21 (16-28)	8.4 (6.1-11)	−13 (−19 to −6.6)
Age 60-69 y	9.5 (7.4-12)	4.8 (3.3-6.9)	−4.8 (−7.3 to −2.3)
Heart failure hospitalization			
Age 50-69 y	14 (12-17)	15 (12-17)	0.1 (−2.6 to 2.9)
Age 50-59 y	12 (8.2-17)	11 (8.8-14)	−1.0 (−5.9 to 3.9)
Age 60-69 y	15 (13-18)	16 (13-19)	0.6 (−2.6 to 3.9)
Stroke			
Age 50-69 y	14 (12-16)	14 (12-16)	0.1 (−2.3 to 2.7)
Age 50-59 y	13 (9.2-18)	12 (9.9-15)	−0.7 (−5.3 to 3.9)
Age 60-69 y	14 (12-16)	15 (12-18)	0.6 (−2.3 to 3.5)

Differences in baseline characteristics between the groups were accounted for by regression standardization. Analyses of the secondary outcomes accounted for the competing risk of death. A detailed description of the statistical methods is available in the Supplemental Appendix.

### All-cause mortality

**Figure 1:**
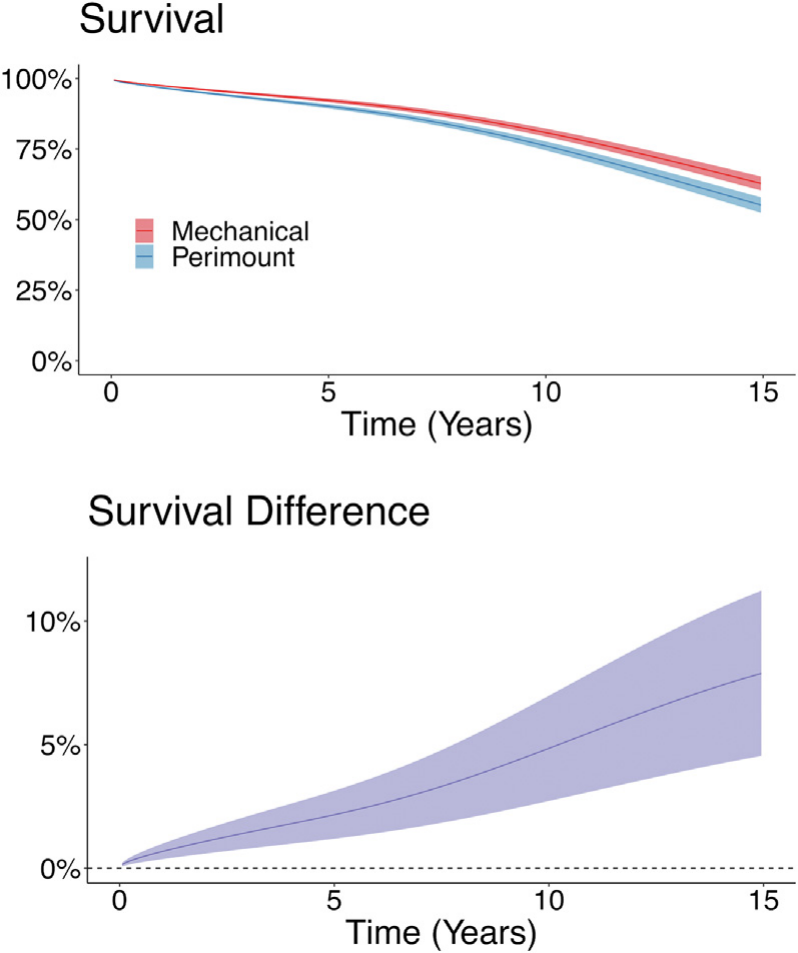
**Survival and Differences in Survival** Regression standardized survival and differences in survival. **(Upper Panel)** The **curves** represent the estimated survival and 95% CI, if the entire population had received a mechanical or a Perimount valve, respectively, eg, if the entire population had received a mechanical valve, the estimated population survival at 15 years would be 63%. **(Lower Panel)** Estimated difference in survival (95% CI) between the mechanical Valve and Perimount groups. Survival was significantly better in the mechanical valve group than in the Perimount group.

During a mean follow-up of 7.4 years (maximum 17.2), 1,370 patients (20%) died. The crude cumulative survival is shown in Supplemental Figure 3. At 15 years of follow-up, the estimated cumulative incidence of all-cause mortality was 37% (95% CI: 35%-40%) in the mechanical valve group and 45% (95% CI: 42%-48%) in the Perimount group. The between-group difference in all-cause mortality at 15 years was −7.9% (95% CI: −11% to −4.6%). The regression standardized cumulative survival is shown in Figure 1.

### Bleeding

**Figure 2:**
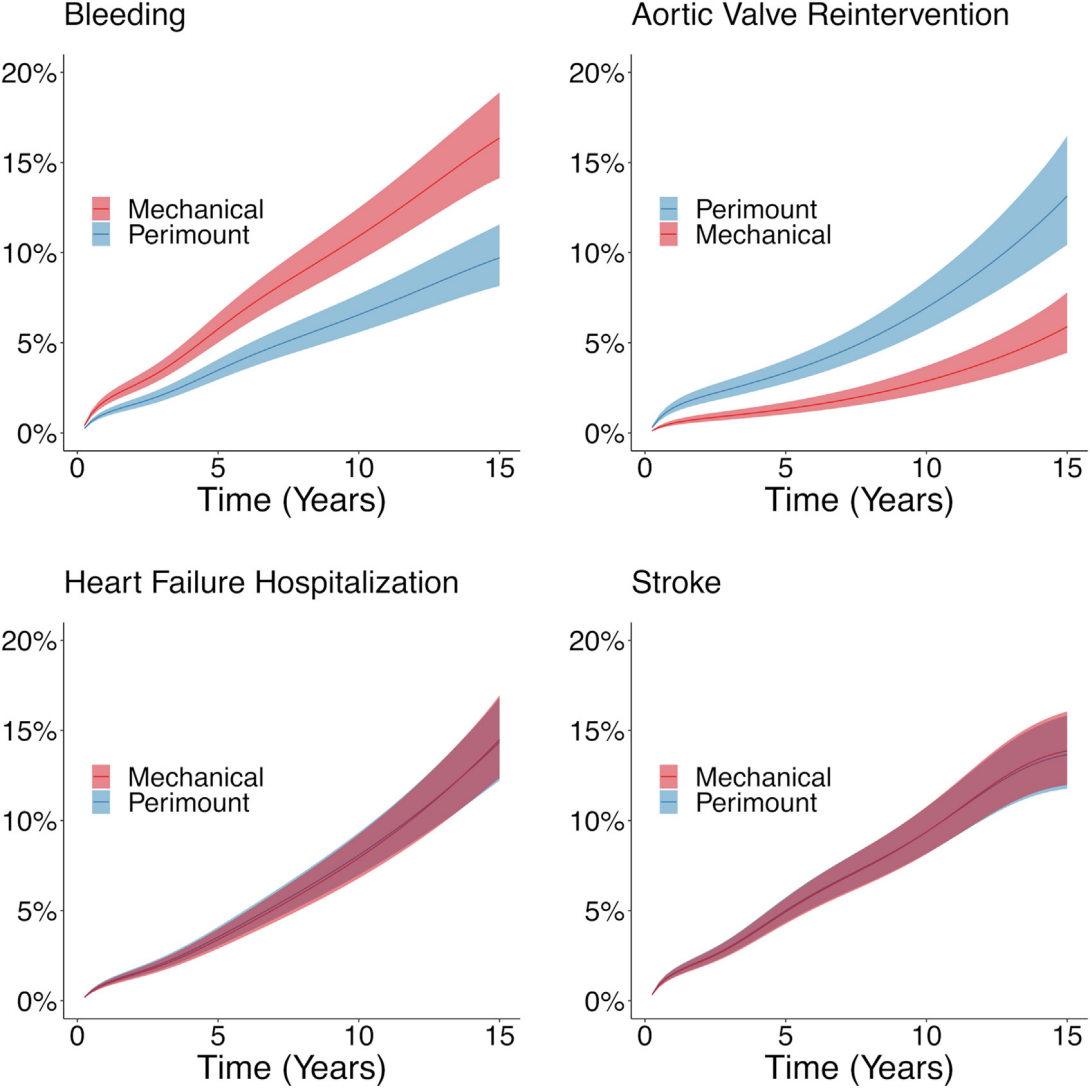
**Cumulative Incidence of Complications** Comparison between regression-standardized cumulative incidence of complications in patients who received a mechanical valve and those who received a Perimount bioprosthesis. The **shaded areas** show the 95% CI. The **curves** represent the estimated cumulative incidence of the respective event, if the entire population had received a mechanical valve or a Perimount valve, respectively. Bleeding was significantly greater and aortic valve reintervention was significantly less common in the mechanical valve group than in the Perimount group. There was no significant difference in the cumulative incidence of heart failure or stroke between the groups (curves superimposed).

During a mean follow-up of 6.1 years (maximum 16.0), 465 patients (6.7%) had a bleeding event. The crude cumulative incidence of bleeding events is shown in Supplemental Figure 4. At 15 years of follow-up, the estimated cumulative incidence of bleeding was 16% (95% CI: 14%-19%) in the mechanical valve group and 9.7% (95% CI: 8.1%-12%) in the Perimount group. The between-group difference in cumulative incidence of bleeding at 15 years was 6.6% (95% CI: 4.1%-9.2%). The regression standardized cumulative incidence of bleeding is shown in Figure 2.

### Aortic valve reintervention

During a mean follow-up of 6.3 years (maximum 16.0), 233 patients (3.4%) underwent aortic valve reintervention (redo AVR or TAVR). The crude cumulative incidence of aortic valve reintervention is shown in Supplemental Figure 4. At 15 years of follow-up, the estimated cumulative incidence of aortic valve reintervention was 5.9% (95% CI: 4.4%-7.8%) in the mechanical valve group and 13% (95% CI: 10%-17%) in the Perimount group. The between-group difference in cumulative incidence of aortic valve reintervention at 15 years was −7.3% (95% CI: −10% to −4.5%). The regression standardized cumulative incidence of aortic valve reintervention is shown in Figure 2.

### Heart Failure hospitalization

During a mean follow-up of 6.2 years (maximum 16.0), 420 patients (6.1%) had a heart failure hospitalization. The crude cumulative incidence of heart failure hospitalization is shown in Supplemental Figure 4. At 15 years of follow-up, the estimated cumulative incidence of heart failure hospitalization was 15% (95% CI: 12%-17%) in the mechanical valve group and 14% (95% CI: 12%-17%) in the Perimount group. The between-group difference in cumulative incidence of heart failure hospitalization at 15 years was 0.1% (95% CI: −2.6% to 2.9%). The regression standardized cumulative incidence of heart failure hospitalization is shown in Figure 2.

### Stroke

During a mean follow-up of 6.1 years (maximum 16.0), 502 patients (7.3%) experienced a stroke event. The crude cumulative incidence of stroke is shown in Supplemental Figure 4. At 15 years of follow-up, the estimated cumulative incidence of stroke was similar in both groups (14%, 95% CI: 12%-16%). The between-group difference in cumulative incidence of stroke was 0.1% (95% CI: −2.3% to 2.7%). The regression standardized cumulative incidence of stroke is shown in Figure 2.

### Sensitivity and subgroup analyses

**Figure 3:**
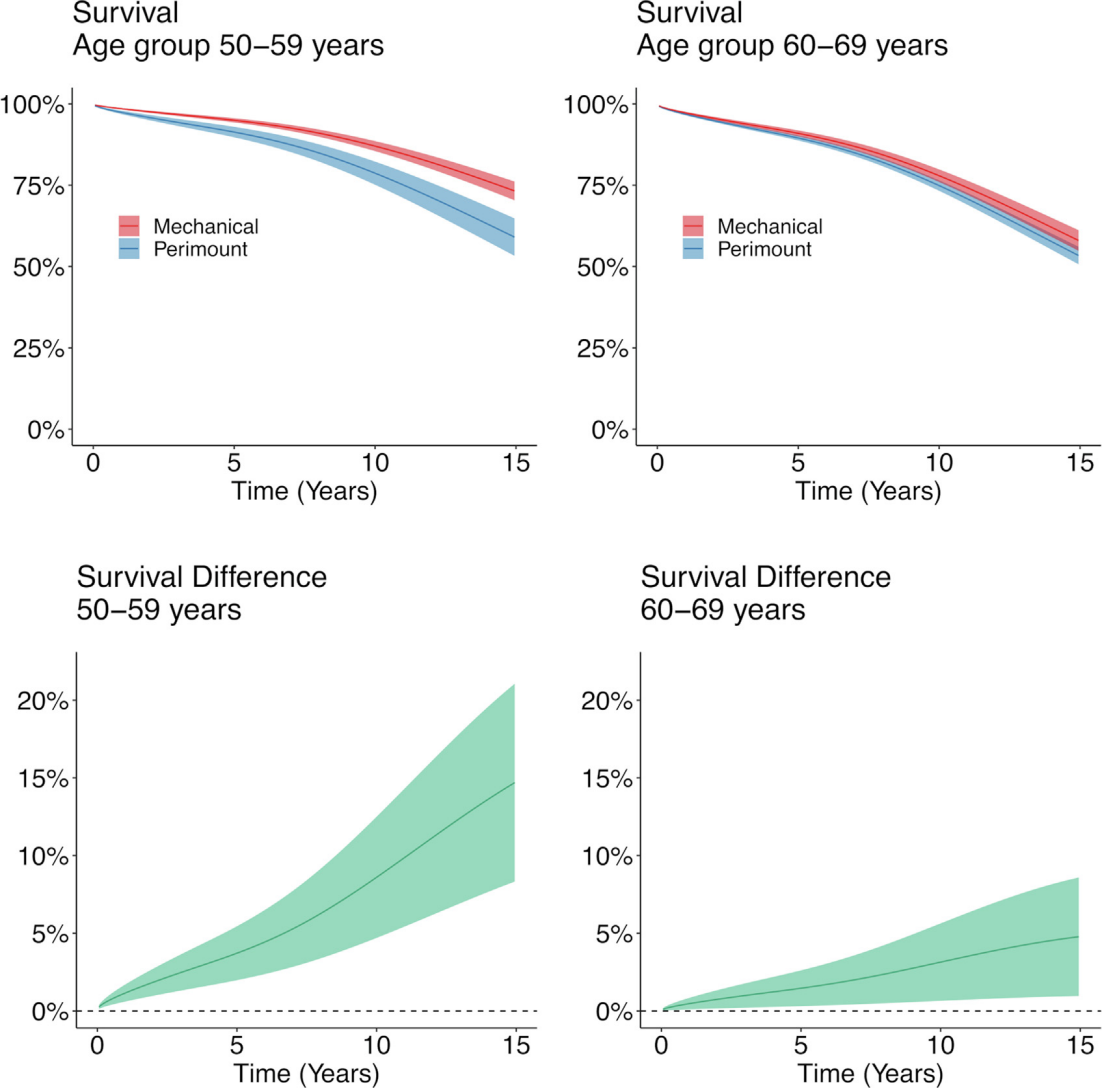
**Survival and Differences in Survival in Age Groups 50 to 59 and 60 to 69 Years** Regression-standardized survival and differences in survival in age groups 50 to 59 years **(left panels)** and 60 to 69 years **(right panels)**. **Upper curves** represent the estimated survival if the entire population had received a mechanical valve or a Perimount valve, respectively. The **lower curves** show the estimated difference in survival between the mechanical valve group and the Perimount group. The **shaded areas** show the 95% CI. Survival was significantly better in the mechanical valve group than in the Perimount group regardless of age group.

When the clinical outcomes were reassessed after stratification according to age group 50 to 59 years or 60 to 69 years, the results were consistent with the findings in the total study population (Central Illustration). The baseline characteristics in the two age groups are shown in Supplemental Tables 6 and 7. The regression standardized survival and survival differences in age groups 50 to 59 years and 60 to 69 years is shown in Table 2 and Figure 3. The regression standardized complication rates are shown in Table 2 and Supplemental Figures 5 and 6. We also conducted analyses using IPTW to control for confounding factors; the results were very similar to those of the main analyses that used regression standardization. All analyses were repeated in the subset of patients who underwent isolated AVR, and the findings were consistent with the findings in the total study population.

**Central Illustration:**
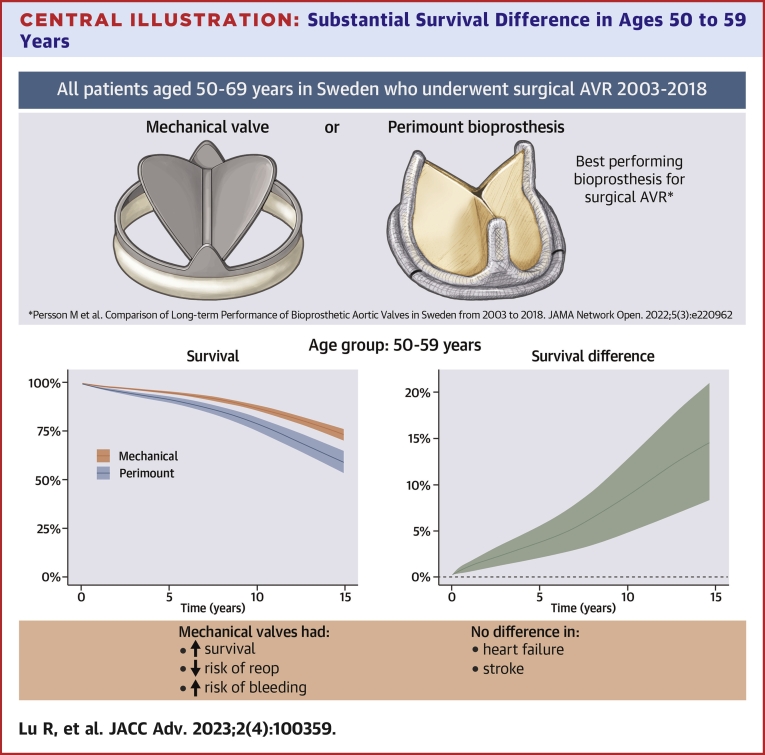
**Substantial Survival Difference in Ages 50 to 59 Years** Can the best bioprosthesis outperform a mechanical valve for surgical AVR in patients aged 50 to 69 years? In 6,907 patients aged 50 to 69 years who underwent surgical AVR 2003 to 2018, survival was better after mechanical vs Perimount bioprosthetic AVR. While valve choice should be guided by individual patient factors and patient preference rather than by chronological age, the substantial survival advantage observed in patients with mechanical valves in ages 50 to 59 years must be recognized. AVR = aortic valve reintervention.

## Discussion

In this study, we found that survival was better after surgical AVR in patients aged 50 to 69 years who received a mechanical valve than in those who received a Perimount bioprosthetic valve. Patients with a mechanical valve had a lower risk of needing aortic valve reintervention but had a higher risk of bleeding complications. No significant between-group difference was found in the risk of hospitalization for heart failure or of stroke. These findings were consistent in patients aged 50 to 59 years and those aged 60 to 69 years. However, in patients aged 60 to 69 years, the absolute survival difference at 15 years was attenuated, while the increased risk of major bleeding remained approximately the same as that in younger patients. While valve choice should be guided by individual patient factors and patient preference rather than by chronological age, the substantial survival advantage observed at the population level in patients with a mechanical valve in the age group 50 to 59 years must be recognized.

Our findings should be interpreted in light of previous studies that investigated clinical outcomes in patients aged 50 to 69 years who underwent surgical AVR with a mechanical valve or a bioprosthesis because the evidence is mixed. Some studies found that long-term survival was significantly better in patients aged 50 to 70 years who received a mechanical valve rather than a bioprosthetic valve,^6^^,^^28^^,^^29^ whereas others found no significant survival difference.^5^^,^^7^ Chiang et al^5^ performed an observational study in patients aged 50 to 69 years, who underwent primary isolated AVR in New York State between 1997 and 2004 and analyzed 1001 propensity score matched patient pairs. Follow-up ended on November 30, 2013, and the authors concluded that there was no significant difference in 15-year survival. Goldstone et al^7^ investigated 9,942 patients who underwent primary, isolated AVR in the state of California between 1996 and 2013 and were followed up until December 31, 2013. After IPTW, the 15-year mortality was significantly higher in patients aged 45 to 54 years who received a bioprosthesis than in those who received a mechanical valve but there was no survival difference in those aged 55 to 64 years. Our previous nationwide study using data from the SWEDEHEART registry included patients aged 50 to 69 years who underwent primary isolated AVR between 1997 and 2013 and were followed up until March 24, 2014. In 1099 propensity score-matched patient pairs, we found that survival was better with a mechanical valve than with a bioprosthetic valve. However, subgroup analyses suggested that the survival benefit was restricted to patients aged 50 to 59 years.^6^ A study from Cleveland Clinic included all patients who underwent isolated AVR from 1990 to January 2020.^30^ Perioperative outcomes were compared in 527 propensity score matched pairs. Early postoperative clinical outcomes were similar between the groups. Adjusted long-term survival in the total study population (5,506 patients with bioprostheses and 637 patients with mechanical valves) was not statistically different between the groups. However, the study did not specifically address outcomes in ages 50 to 69 years, and there was a clear institutional preference for bioprosthetic rather than mechanical valves. Moreover, this was a tertiary referral single center experience, and it is unclear to what extent the results are generalizable to other institutions.

### Complications after AVR

The findings of previous studies have been largely concordant regarding complications following AVR and concluded that patients who received a mechanical valve have a higher cumulative incidence of bleeding but a lower risk of reoperation.^31^ Notably, Goldstone et al concluded that bioprostheses were associated with fewer strokes in patients aged 45 to 54 years, while the studies by Chiang et al and Glaser et al found no difference in stroke rate between the mechanical and bioprosthesis groups.^5^, ^6^, ^7^

Our results support the current European Society of Cardiology/European Association for Cardio-Thoracic Surgery recommendation that a mechanical prosthesis should be considered for the aortic position in patients aged younger than 60 years.^9^ The American College of Cardiology/American Heart Association guideline for the management of valvular heart disease^8^ favors a mechanical aortic valve over a bioprosthetic valve for patients aged younger than 50 years. However, this recommendation is challenged by our observation of an absolute survival difference of 15% at 15 years between patients with a mechanical valve and those with a Perimount bioprosthesis in the age group 50 to 59 years. It is important to recognize that the Perimount valve has demonstrated superior performance and that the survival difference may be even larger when some of the other bioprosthetic valve models are used in this age group.^10^^,^^11^^,^^32^

### Study Strengths and limitations

This study has several strengths. We were able to link information from several high-quality and complete nationwide health-data registers in Sweden, which allowed careful characterization of the study population, including demographics, medical history, comorbidities, and socioeconomic status. We identified the most used and best performing bioprosthetic valve model because results obtained from studies using obsolete valve models are less useful. Generalizability was high because of the recent study period and the population-based design, which included all hospitals performing cardiac surgery in Sweden. Moreover, we included patients who underwent AVR in combination with coronary artery bypass surgery or ascending aortic repair, because surgical AVR is often performed in addition to other cardiac surgical procedures, and valve choice is relevant also in those populations. There were very small amounts of missing baseline data, and no patients were lost to follow-up; therefore, data regarding the main outcome measure can be considered complete. The statistical analyses were designed and conducted to allow estimation of absolute effect measures that help quantify the risk or benefit associated with each valve type.

Our study also has some limitations. First, we were not able to ascertain secondary outcomes (aortic valve reintervention, major bleeding events, heart failure hospitalization, or stroke) that occurred outside of Sweden. However, in view of the universal tax-financed health care coverage in Sweden, these were likely to have been infrequent, and it is reasonable to assume that these events occurred nondifferentially between the groups. Therefore, this limitation may lead to underestimation of event rates but is unlikely to bias the results. Deaths outside of Sweden were captured by the Population Register, and follow-up regarding the main outcome was thus complete.^20^ Second, although our characterization of the study population was granular, we lacked information regarding potentially important patient features (eg, frailty) that would be associated with both exposure (valve choice) and outcome (all-cause mortality). Thus, residual confounding could have affected our findings. Third, three Perimount models (2900, Magna [3000], and Magna Ease [3300]) were categorized into 1 group. It is possible that performance may differ between these three models.^32^ Fourth, the Swedish national health-data registers do not contain echocardiographic data. Therefore, we could not ascertain structural valve deterioration according to current definitions^33^^,^^34^ and relied on surrogate measures (aortic valve reintervention and heart failure hospitalization).

## Conclusions

We found that in patients aged 50 to 69 years who underwent surgical AVR, survival was better in those who received a mechanical valve than in those who received a Perimount bioprosthesis. Patients with a mechanical valve had less risk of aortic valve reintervention but a higher risk of bleeding complications. The survival advantage associated with a mechanical valve was substantial in patients aged 50 to 59 years, suggesting that it may be prudent to adjust the current American College of Cardiology/American Heart Association valvular heart disease guideline to recommend a mechanical valve for patients up to 60 years of age.

PERSPECTIVES **COMPETENCY IN MEDICAL KNOWLEDGE:** Evidence is mixed regarding the most appropriate type of valve prosthesis for surgical AVR in patients 50 to 69 years and American and European guideline recommendations differ.

**COMPETENCY IN PATIENT CARE:** In patients aged 50 to 69 years who underwent surgical AVR in Sweden, survival was better in those who received mechanical compared to Perimount bioprosthetic valves, and a substantial survival advantage was observed in ages 50 to 59 years.

**TRANSLATIONAL OUTLOOK:** Patients with mechanical valves had better survival and a lower risk of aortic valve reintervention, but a higher risk for bleeding. Better strategies to optimize anticoagulation treatment in patients with a mechanical heart valve are needed.

## Funding support and author disclosures

This work was supported by the 10.13039/501100003793Swedish Heart-Lung Foundation (grant number 20190570 to NG and grant number 20190533 to US), Region Stockholm (ALF Project) (grant number FoUI-954783 and FoUI-961871 to Dr Glaser and grant number FoUI-962048 to Dr Sartipy), Region Stockholm clinical postdoctoral appointment (FoUI-955489 to Dr Glaser), the 10.13039/501100007687Swedish Society of Medicine (grant number SLS-934749 to Dr Glaser), Eva and Oscar Ahrén Research Foundation (to Dr Glaser), the 10.13039/100018876Seraphim Hospital Foundation (to Dr Glaser), 10.13039/501100006285Magnus Bergvall Foundation (grant number 2021-04333 to Dr Glaser), and Mats 10.13039/100012394Kleberg Foundation (grant number 2022-119 to Dr Glaser). All other authors have reported that they have no relationships relevant to the contents of this paper to disclose.
