# Supplementary material for: Aortic Valve Replacement With Mechanical Valves vs Perimount Bioprostheses in 50- to 69-Year-Old Patients
Source: JACC Adv. 2023 Jun 7;2(4):100359. doi: 10.1016/j.jacadv.2023.100359 (PMC11198651; doi:10.1016/j.jacadv.2023.100359)
Supplement: Supplementary data [file mmc1.docx]

Supplemental Appendix

# Supplemental Statistical Methods

*Regression standardization*

In survival analysis, regression standardization helps researchers obtain a direct standardized survival curve. The method for this analysis is as follows. First, the regression model is run with the exposure, outcome and potential confounders as covariates. The exposure is then recoded for all patients in the population to true. This model is then used to predict the effect measure for each of the patients in the population, and finally, the average these predictions. This procedure is repeated, but the exposure is set to the next level (or in this example with a binary exposure, the exposure is set to false). In this example, the method will provide two survival curves of one for exposed, and one for non-exposed. Each curve represents the hypothetical population where all patients either received or did not receive the treatment, standardized by (i.e., adjusted for) the population distribution of covariates. The results are intuitive and can be interpreted as follows. If the entire population received treatment X, 70% would be alive at 10 years. If the entire population received treatment Y instead, only 50% would be alive at 10 years. Regression standardization answers what in causal inference often is referred to as a counterfactual question, characterized by its “if instead…” nature. In fact, if measured covariates are sufficient for confounder control, these results can be interpreted as causal effects for the population under study. For further reading, we recommend Sjölander 2016, Kipouro 2019 and Rothman 2012 p.475-478, 518-521.^1-3^

*Model selection*

We used a model selection strategy that combined subject matter knowledge and backward selection using all variables in Table 1. The final model selection was informed using the Akaike information criterion (AIC). Continuous covariates were tested using splines and polynomials. We explored time dependent effects by including interaction terms with the base-line hazard as described for each model below.

*Survival*

The covariates included in the final model were: valve type, categorical age, centered age, the cubic centered age, the operating hospital, sex, left ventricular ejection fraction, ascending aortic surgery, birth region, education level, prior myocardial infarction, history of cancer, chronic obstructive pulmonary disease, diabetes mellitus, prior endocarditis, prior heart failure, hyperlipidemia, hypertension, hepatic disease, hypertension, peripheral vascular disease, prior stroke, prior pacemaker or implantable cardioversion device, prior percutaneous coronary intervention, marital status, categorical household income, categorical body mass index, period of surgery, categorical estimated glomerular filtration rate, categorical size of prostheses, prior major bleeding event, and emergent operation. The baseline hazard was modeled using a natural spline with three degrees of freedom. In interaction between valve type and categorical age was included.

*Bleeding*

The final model for bleeding used a 3rd degree exponential b-spline with 2 inner knots at the 1/3 and 2/3 quantiles for the baseline hazard. Age was centered and included as a linear term. An interaction between valve type and categorical age was included. Polynomial conversion or splines of age did not improve model performance. The covariates included were: valve model, categorical age, centered age, operating hospital, sex, left ventricular ejection fraction, concomitant coronary artery bypass grafting, ascending aortic surgery, birth region, education, prior myocardial infarction, history of cancer, chronic obstructive pulmonary disease, diabetes mellitus, prior endocarditis, prior heart failure, hyperlipidemia, hypertension, hepatic disease, peripheral vascular disease, prior stroke, prior pacemaker or implantable cardioversion device, prior percutaneous coronary intervention, marital status, categorical household income, categorical body mass index, period of surgery, categorical estimated glomerular filtration rate, valve size, and prior major bleeding event.

*Aortic valve reintervention*

For reintervention the final model used a 3rd degree exponential b-spline with 2 inner knots at the 1/3 and 2/3 quantiles for the baseline hazard. Age was centered and included as a linear term. An interaction term between centered age and valve type and the baseline hazard was also included. The covariates included were: valve model, categorical age, centered age, the cubic centered age, operating hospital, sex, left ventricular ejection fraction, concomitant coronary artery bypass grafting, ascending aortic surgery, birth region, education, prior myocardial infarction, history of cancer, chronic obstructive pulmonary disease, diabetes mellitus, prior endocarditis, prior heart failure, hyperlipidemia, hypertension, hepatic disease, hypertension, peripheral vascular disease, prior stroke, prior pacemaker or implantable cardioversion device, prior percutaneous coronary intervention, marital status, categorical household income, categorical body mass index, period of surgery, categorical estimated glomerular filtration rate, and valve size.

*Heart failure hospitalization*

The final model for rehospitalization used a 3rd degree exponential b-spline with 2 inner knots at the 1/3 and 2/3 quantiles for the baseline hazard. Age was centered and included as a linear term. An interaction between valve type and categorical age was included. Polynomial conversion or splines of age did not improve model performance. The covariates included were: valve model, categorical age, centered age, operating hospital, sex, left ventricular ejection fraction, concomitant coronary artery bypass grafting, ascending aortic surgery, birth region, education, prior myocardial infarction, history of cancer, chronic obstructive pulmonary disease, diabetes mellitus, prior endocarditis, prior heart failure, hyperlipidemia, hypertension, hepatic disease, peripheral vascular disease, prior stroke, prior pacemaker or implantable cardioversion device, prior percutaneous coronary intervention, marital status, categorical household income, categorical body mass index, period of surgery, categorical estimated glomerular filtration rate, and valve size.

*Stroke*

The final model for stroke used a 3rd degree exponential b-spline with 2 inner knots at the 1/3 and 2/3 quantiles for the baseline hazard. Age was centered and included as a linear term. An interaction between valve type and categorical age was included. Polynomial conversion or splines of age did not improve model performance. The covariates included were: valve model, categorical age, centered age, operating hospital, sex, left ventricular ejection fraction, concomitant coronary artery bypass grafting, ascending aortic surgery, birth region, education, prior myocardial infarction, history of cancer, chronic obstructive pulmonary disease, diabetes mellitus, prior endocarditis, prior heart failure, hyperlipidemia, hypertension, hepatic disease, peripheral vascular disease, prior stroke, prior pacemaker or implantable cardioversion device, prior percutaneous coronary intervention, marital status, categorical household income, categorical body mass index, period of surgery, categorical estimated glomerular filtration rate, valve size, prior major bleeding event, and emergent operation.

***References***

1. Sjölander A. Regression standardization with the R package stdReg. Eur J Epidemiol. 2016;31(6):563-74.

2. Kipourou DK, Charvat H, Rachet B, Belot A. Estimation of the adjusted cause-specific cumulative probability using flexible regression models for the cause-specific hazards. Stat Med. 2019;38(20):3896-3910.

3. Rothman KJ, Lash TL, VanderWeele TJ, Haneuse S. Modern epidemiology. Fourth ed. Wolters Kluwer; 2021.

| Supplemental Table 1. Frequencies of mechanical and Perimount valve models. | |
| --- | --- |
| Model | No. |
| Mechanical valve group | |
| Carbomedics Reduced Aortic R5 | 1022 |
| Aortic HP Masters mechanic heart valve AHPJ-505 | 314 |
| Regent aortic MHV AGN-751 | 255 |
| On-X Aortic | 216 |
| Carbomedics Carbo-Seal AP | 179 |
| Carbomedics SERIALNO | 148 |
| Aortic Masters Series | 145 |
| Regent aortic MHV flex cuff AGFN-756 | 114 |
| ATS Open Pivot Standard Heart Valve 500FA | 104 |
| Bicarbon Slimline Aortic LSA | 93 |
| Carbomedics Carbo-Seal Valsalva CP | 87 |
| Carbomedics Top Hat Supraannular Aortic S5 | 70 |
| Masters series CAVG compositegraft CAVGJ-514 00 | 60 |
| St. Jude Mechanical Aortic Standard A-101 | 49 |
| ATS Open Pivot AP Series Heart Valve 505DA | 43 |
| ATS Open Pivot AP Series Heart Valve 501DA | 32 |
| Masters Series VAVGJ Composite | 30 |
| St. Jude Composite Aortic Valved Graft Unknown | 25 |
| Carbomedics Standard Aortic A5 | 19 |
| Medtronic Hall Aortic | 18 |
| Masters Standard Aortic mechanic heart valve AJ-501 | 14 |
| ATS Open Pivot Aortic Valved Graft (AVG) 502AG | 8 |
| Edwards Mira Mechanical Valve | 7 |
| Bicarbon Aortic LN | 6 |
| Other mechanical valve | 6 |
| On-X Aortic SERIALNO | 5 |
| ATS Open Pivot AP series Heart Valve AP360 | 3 |
| Ultracor Tilting Disc Heart Valve Prosthesis | 1 |
| Omnicarbon Aortic Valve | 1 |
| Bicarbon Overline Aortic LOV | 1 |
| Masters Series MTJ-503 | 1 |
| Perimount bioprostheses | |
| Perimount 2900 | 2964 |
| Perimount Magna Ease 3300TFX | 664 |
| Perimount Magna 3000 | 107 |
| Not specified | 96 |
|  | |

| Supplemental Table 2. ICD codes for secondary outcomes. | |
| --- | --- |
| Outcome | ICD codes |
| Aortic valve reintervention | FMD, FCA60, FCA70 |
| Bleeding event | I60, I61, I62, I850, K226, K250, K252, K254, K256, K260, K262, K264, K266, K270, K272, K274, K276, K280, K282, K284, K286, K290, K625, K920, K921, K922, D629, D500, R040, R041, R042, R048, R049, R319, I312, J942 |
| Heart failure | I50 |
| Stroke | I63, I64, G458, G459, I74 |
| ICD = International Classification of Diseases | |

| Supplemental Table 3. Incidence rates of all-cause mortality, bleeding, reintervention, heart failure hospitalization and stroke after surgical aortic valve replacement with a Perimount or mechanical valve in patients aged 50–69. | | |
| --- | --- | --- |
| Outcome | Perimount | Mechanical |
| All-cause mortality | | |
| No. of events | 752 | 618 |
| Crude | 3.1 (2.9–3.3) | 2.3 (2.1–2.5) |
| Age- and sex-adjusted | 3.0 (2.8–3.2) | 2.8 (2.6–3.0) |
| Bleeding | | |
| No. of events | 183 | 282 |
| Crude | 0.92 (0.79–1.07) | 1.26 (1.12–1.41) |
| Age- and sex-adjusted | 0.87 (0.76–0.99) | 1.34 (1.14–1.57) |
| Aortic valve reintervention | | |
| No. of events | 144 | 89 |
| Crude | 0.72 (0.61–0.85) | 0.38 (0.31–0.47) |
| Age- and sex-adjusted | 0.93 (0.75–1.14) | 0.36 (0.30–0.42) |
| Heart failure hospitalization | | |
| No. of events | 218 | 202 |
| Crude | 1.10 (0.96–1.25) | 0.88 (0.76–1.01) |
| Age- and sex-adjusted | 1.01 (0.87–1.17) | 1.06 (0.93–1.22) |
| Stroke | | |
| No. of events | 254 | 248 |
| Crude | 1.31 (1.15–1.48) | 1.10 (0.97–1.24) |
| Age- and sex-adjusted | 1.22 (1.05–1.40) | 1.19 (1.03–1.36) |
| Incidence rate = number of events per person-year of follow-up. Age- and sex-adjusted incidence rates were obtained from a Poisson model. CI = confidence interval. | | |

| Supplemental Table 4. Regression standardized cumulative incidence at 5 years of all-cause mortality, bleeding events, aortic valve reintervention, heart failure hospitalization, and stroke after surgical aortic valve replacement with a Perimount or mechanical valve in patients aged 50–69 years, 50–59 years and 60–69 years. | | | |
| --- | --- | --- | --- |
|  | Cumulative incidence (95% CI) | | Cumulative incidence  difference (95% CI) |
|  | Perimount | Mechanical | Mechanical vs. Perimount |
| All-cause mortality | | | |
| Age 50-69 | 9.9 (9.2–11) | 7.8 (7.1–8.5) | -2.2 (-3.1– -1.2) |
| *Age 50-59* | *8.7 (7.1–10)* | *5.1 (4.4–5.7)* | *-3.7 (-5.5 – -2.0)* |
| *Age 60-69* | *11 (9.7–11)* | *9.1 (8.2–10)* | *-1.5 (-2.6 – -0.3)* |
| Bleeding |  |  |  |
| Age 50-69 | 3.5 (3.0–4.1) | 5.8 (5.0–6.6) | 2.3 (1.3–3.2) |
| *Age 50-59* | *2.7 (1.7–4.1)* | *4.9 (4.1–5.9)* | *2.3 (0.8–3.7)* |
| *Age 60-69* | *3.8 (3.3–4.5)* | *6.1 (5.2–7.3)* | *2.3 (1.1–3.5)* |
| Aortic valve reintervention | | | |
| Age 50-69 | 3.3 (2.8–4.1) | 1.3 (1.0–1.7) | 2.0 (-2.7– -1.3) |
| *Age 50-59* | *5.4 (4.0–7.3)* | *1.8 (1.3–2.4)* | *-3.7 (-5.3– -2)* |
| *Age 60-69* | *2.4 (1.9–2.9)* | *1.1 (0.8–1.6)* | *-1.3 (-1.9– -0.7)* |
| Heart failure hospitalization | | | |
| Age 50-69 | 3.5 (3.0–4.1) | 3.4 (2.9–4.0) | -0.1 (-0.9–0.6) |
| *Age 50-59* | *2.7 (1.9–4.1)* | *2.3 (1.8–2.9)* | *-0.4 (-1.6–0.7)* |
| *Age 60-69* | *3.9 (3.4–4.5)* | *3.9 (3.2–4.7)* | *0.0 (-0.9–0.9)* |
| Stroke |  |  |  |
| Age 50-69 | 5.0 (4.4–5.7) | 4.9 (4.3–5.7) | -0.1 (-1.0–0.9) |
| *Age 50-59* | *4.5 (3.2–6.3)* | *4.0 (3.3–4.9)* | *-0.5 (-2.2–1.2)* |
| *Age 60-69* | *5.2 (4.6–6.0)* | *5.3 (4.5–6.4)* | *0.1 (-1.0–1.3)* |
| Numbers are percentages. CI = confidence interval. Differences in baseline characteristics between the groups were accounted for by regression standardization. Analyses of the secondary outcomes accounted for the competing risk of death. | | | |

| Supplemental Table 5. Regression standardized cumulative incidence at 10 years of all-cause mortality, bleeding events, aortic valve reintervention, heart failure hospitalization, and stroke after surgical aortic valve replacement with a Perimount or mechanical valve in patients aged 50–69 years, 50–59 years and 60–69 years. | | | |
| --- | --- | --- | --- |
|  | Cumulative incidence (95% CI) | | Cumulative incidence  difference (95% CI) |
|  | Perimount | Mechanical | Mechanical vs. Perimount |
| *All-cause mortality* | | | |
| Age 50-69 | 24 (22–26) | 19 (18–21) | -4.9 (-7.0– -2.7) |
| *Age 50-59* | *21 (18–25)* | *13 (11–15)* | *-8.6 (-13– -4.7)* |
| *Age 60-69* | *25 (24–27)* | *22 (20–24)* | *-3.1 (-5.6– -0.6)* |
| Bleeding |  |  |  |
| Age 50-69 | 6.6 (5.6–7.7) | 11 (9.5–13) | 4.4 (2.6–6.1) |
| *Age 50-59* | *5.1 (3.3–7.8)* | *9.5 (8.0–11)* | *4.5 (1.7–7.2)* |
| *Age 60-69* | *7.2 (6.2–8.4)* | *12 (9.8–14)* | *4.3 (2.2–6.4)* |
| Aortic valve reintervention | | | |
| Age 50-69 | 7.0 (5.7–8.4) | 2.9 (2.2–3.7) | -4.1 (-5.5– -2.7) |
| *Age 50-59* | *11.2 (8.4–15)* | *3.9 (3.0–5.3)* | *-7.3 (-11– -3.9)* |
| *Age 60-69* | *5.0 (4.0–6.2)* | *2.4 (1.7–3.4)* | *-2.6 (-3.9– -1.3)* |
| Heart failure hospitalization | | | |
| Age 50-69 | 8.1 (7.0–9.3) | 7.9 (6.8–9.2) | -0.2 (-1.8–1.4) |
| *Age 50-59* | *6.4 (4.4–9.4)* | *5.6 (4.5–7.0)* | *-0.8 (-3.5–1.9)* |
| *Age 60-69* | *8.8 (7.7–10)* | *9.0 (7.5–11)* | *0.2 (-1.8–2.1)* |
| Stroke |  |  |  |
| Age 50-69 | 9.4 (8.2–11) | 9.4 (8.1–11) | 0.0 (-1.8–1.7) |
| *Age 50-59* | *8.6 (6.2–12)* | *7.9 (6.5–9.5)* | *-0.7 (-3.9–2.4)* |
| *Age 60-69* | *9.7 (8.5–11)* | *10 (8.5–12)* | *0.3 (-1.7–2.4)* |
| Numbers are percentages. CI = confidence interval. Differences in baseline characteristics between the groups were accounted for by regression standardization. Analyses of the secondary outcomes accounted for the competing risk of death. | | | |

| Supplemental Table 6. Baseline characteristics in patients aged 50–59 years who underwent surgical aortic valve replacement with a Perimount or mechanical valve prosthesis. | | | |
| --- | --- | --- | --- |
| Variable | Overall | Perimount | Mechanical |
| No. | 2164 | 573 | 1591 |
| Age, mean(SD), years | 55.5 (2.8) | 55.9 (2.7) | 55.3 (2.8) |
| Male sex | 1641 (75.8) | 433 (75.6) | 1208 (75.9) |
| Married | 1161 (53.7) | 276 (48.2) | 885 (55.6) |
| Education |  |  |  |
| <10 years | 552 (25.6) | 131 (22.9) | 421 (26.6) |
| 10-12 years | 1070 (49.6) | 290 (50.8) | 780 (49.2) |
| >12 years | 534 (24.8) | 150 (26.3) | 384 (24.2) |
| Non-Nordic birth region | 211 ( 9.8) | 66 (11.5) | 145 ( 9.1) |
| Household income |  |  |  |
| Q1 (lowest) | 346 (16.0) | 130 (22.7) | 216 (13.6) |
| Q2 | 422 (19.5) | 105 (18.3) | 317 (19.9) |
| Q3 | 548 (25.3) | 110 (19.2) | 438 (27.5) |
| Q4 (highest) | 848 (39.2) | 228 (39.8) | 620 (39.0) |
| Body mass index, kg/m^2^ |  |  |  |
| <18.5 | 16 (0.8) | 5 (0.9) | 11 (0.8) |
| 18.5-24.9 | 567 (28.3) | 142 (26.4) | 425 (29.0) |
| 25-29.9 | 866 (43.3) | 239 (44.4) | 627 (42.9) |
| >=30 | 552 (27.6) | 152 (28.3) | 400 (27.3) |
| Diabetes mellitus | 292 (13.5) | 86 (15.0) | 206 (12.9) |
| Prior atrial fibrillation | 201 (9.3) | 57 (9.9) | 144 (9.1) |
| Hypertension | 828 (38.3) | 241 (42.1) | 587 (36.9) |
| Hyperlipidemia | 377 (17.4) | 87 (15.2) | 290 (18.2) |
| Prior stroke | 138 (6.4) | 51 (8.9) | 87 (5.5) |
| Peripheral vascular disease | 337 (15.6) | 87 (15.2) | 250 (15.7) |
| COPD | 127 (5.9) | 47 (8.2) | 80 (5.0) |
| Prior myocardial infarction | 223 (10.3) | 63 (11.0) | 160 (10.1) |
| Prior PCI | 111 (5.1) | 40 (7.0) | 71 (4.5) |
| Pacemaker/ICD | 36 (1.7) | 12 (2.1) | 24 (1.5) |
| Prior major bleeding event | 100 (4.6) | 46 (8.0) | 54 (3.4) |
| Alcohol dependence | 113 (5.2) | 49 (8.6) | 64 (4.0) |
| Hepatic disease | 41 (1.9) | 23 (4.0) | 18 (1.1) |
| History of cancer | 120 (5.5) | 37 (6.5) | 83 (5.2) |
| eGFR, mL/min/1.73m^2^ |  |  |  |
| <30 | 52 (2.5) | 21 (3.7) | 31 (2.0) |
| 30-44 | 22 (1.0) | 11 (2.0) | 11 (0.7) |
| 45-59 | 93 (4.4) | 28 (5.0) | 65 (4.2) |
| >=60 | 1946 (92.1) | 503 (89.3) | 1443 (93.1) |
| Prior heart failure | 343 (15.9) | 87 (15.2) | 256 (16.1) |
| Prior endocarditis | 213 ( 9.8) | 74 (12.9) | 139 (8.7) |
| LVEF |  |  |  |
| <30% | 113 (5.3) | 34 (6.0) | 79 (5.0) |
| 30-50% | 425 (19.9) | 110 (19.5) | 315 (20.0) |
| >50% | 1603 (74.9) | 421 (74.5) | 1182 (75.0) |
| Emergent operation | 50 (2.3) | 22 (3.9) | 28 (1.8) |
| Isolated AVR | 1361 (62.9) | 389 (67.9) | 972 (61.1) |
| Concomitant CABG | 328 (15.2) | 83 (14.5) | 245 (15.4) |
| Ascending aortic surgery | 532 (24.6) | 111 (19.4) | 421 (26.5) |
| Valve size, mm |  |  |  |
| 18-21 | 415 (19.4) | 103 (18.0) | 312 (20.0) |
| 22-23 | 762 (35.7) | 187 (32.6) | 575 (36.8) |
| 24-29 | 958 (44.9) | 283 (49.4) | 675 (43.2) |
| Period of surgery, years |  |  |  |
| 2003-2008 | 760 (35.1) | 109 (19.0) | 651 (40.9) |
| 2009-2013 | 652 (30.1) | 204 (35.6) | 448 (28.2) |
| 2014-2018 | 752 (34.8) | 260 (45.4) | 492 (30.9) |
| Numbers are N and (%) unless otherwise stated. SD = standard deviation, AVR = aortic valve replacement, CABG = coronary artery bypass graft, COPD = chronic obstructive pulmonary disease, eGFR = estimated glomerular filtration rate, ICD = implantable cardioverter-defibrillator, LVEF = left ventricular ejection fraction, PCI = percutaneous coronary intervention, Q = quartile. | | | |

| Supplemental Table 7. Baseline characteristics in patients aged 60–69 years who underwent surgical aortic valve replacement with a Perimount or mechanical valve prosthesis. | | | |
| --- | --- | --- | --- |
| Variable | Overall | Perimount | Mechanical |
| No. | 4743 | 3258 | 1485 |
| Age, mean(SD), years | 64.9 (2.8) | 65.5 (2.7) | 63.6 (2.6) |
| Male sex | 3445 (72.6) | 2358 (72.4) | 1087 (73.2) |
| Married | 3004 (63.3) | 2048 (62.9) | 956 (64.4) |
| Education |  |  |  |
| <10 years | 1613 (34.2) | 1094 (33.8) | 519 (35.3) |
| 10-12 years | 1983 (42.1) | 1357 (41.9) | 626 (42.6) |
| >12 years | 1115 (23.7) | 789 (24.4) | 326 (22.2) |
| Non-Nordic birth region | 307 (6.5) | 186 (5.7) | 121 (8.1) |
| Household income |  |  |  |
| Q1 (lowest) | 709 (15.0) | 486 (14.9) | 223 (15.0) |
| Q2 | 937 (19.8) | 626 (19.2) | 311 (20.9) |
| Q3 | 1266 (26.7) | 838 (25.7) | 428 (28.8) |
| Q4 (highest) | 1830 (38.6) | 1307 (40.1) | 523 (35.2) |
| Body mass index, kg/m^2^ |  |  |  |
| <18.5 | 34 (0.8) | 28 (0.9) | 6 (0.4) |
| 18.5-24.9 | 1306 (29.1) | 912 (29.2) | 394 (28.8) |
| 25-29.9 | 1915 (42.7) | 1328 (42.5) | 587 (43.0) |
| >=30 | 1235 (27.5) | 856 (27.4) | 379 (27.7) |
| Diabetes mellitus | 987 (20.8) | 723 (22.2) | 264 (17.8) |
| Prior atrial fibrillation | 708 (14.9) | 442 (13.6) | 266 (17.9) |
| Hypertension | 2464 (52.0) | 1805 (55.4) | 659 (44.4) |
| Hyperlipidemia | 1093 (23.0) | 787 (24.2) | 306 (20.6) |
| Prior stroke | 435 (9.2) | 322 (9.9) | 113 (7.6) |
| Peripheral vascular disease | 719 (15.2) | 469 (14.4) | 250 (16.8) |
| COPD | 499 (10.5) | 354 (10.9) | 145 (9.8) |
| Prior myocardial infarction | 592 (12.5) | 414 (12.7) | 178 (12.0) |
| Prior PCI | 366 (7.7) | 271 (8.3) | 95 (6.4) |
| Pacemaker/ICD | 98 (2.1) | 67 (2.1) | 31 (2.1) |
| Prior major bleeding event | 334 ( 7.0) | 268 (8.2) | 66 (4.4) |
| Alcohol dependence | 189 ( 4.0) | 152 (4.7) | 37 (2.5) |
| Hepatic disease | 81 ( 1.7) | 67 (2.1) | 14 (0.9) |
| History of cancer | 508 (10.7) | 380 (11.7) | 128 (8.6) |
| eGFR, mL/min/1.73m^2^ |  |  |  |
| <30 | 104 ( 2.2) | 78 (2.4) | 26 (1.8) |
| 30-44 | 131 ( 2.8) | 101 (3.2) | 30 (2.1) |
| 45-59 | 497 (10.7) | 362 (11.3) | 135 (9.3) |
| >=60 | 3922 (84.3) | 2656 (83.1) | 1266 (86.9) |
| Prior heart failure | 899 (19.0) | 627 (19.2) | 272 (18.3) |
| Prior endocarditis | 262 ( 5.5) | 195 (6.0) | 67 (4.5) |
| LVEF |  |  |  |
| <30% | 285 ( 6.0) | 204 (6.3) | 81 (5.5) |
| 30-50% | 932 (19.8) | 634 (19.6) | 298 (20.2) |
| >50% | 3495 (74.2) | 2401 (74.1) | 1094 (74.3) |
| Emergent operation | 86 ( 1.8) | 57 (1.8) | 29 (2.0) |
| Isolated AVR | 2769 (58.4) | 1942 (59.6) | 827 (55.7) |
| Concomitant CABG | 1313 (27.7) | 920 (28.2) | 393 (26.5) |
| Ascending aortic surgery | 805 (17.0) | 478 (14.7) | 327 (22.0) |
| Valve size, mm |  |  |  |
| 18-21 | 1085 (23.0) | 738 (22.7) | 347 (23.7) |
| 22-23 | 1806 (38.3) | 1260 (38.8) | 546 (37.3) |
| 24-29 | 1820 (38.6) | 1248 (38.4) | 572 (39.0) |
| Period of surgery, years |  |  |  |
| 2003-2008 | 1428 (30.1) | 668 (20.5) | 760 (51.2) |
| 2009-2013 | 1514 (31.9) | 1106 (33.9) | 408 (27.5) |
| 2014-2018 | 1801 (38.0) | 1484 (45.5) | 317 (21.3) |
| Numbers are N and (%) unless otherwise stated. SD = standard deviation, AVR = aortic valve replacement, CABG = coronary artery bypass graft, COPD = chronic obstructive pulmonary disease, eGFR = estimated glomerular filtration rate, ICD = implantable cardioverter-defibrillator, LVEF = left ventricular ejection fraction, PCI = percutaneous coronary intervention, Q = quartile. | | | |

Supplemental Figure 1. Number of aortic valve replacements per year in patients aged 50–69 years. Red bars represent the number of mechanical valves, and blue bars represent the number of Perimount valves in Sweden between 2003 to 2018.

**
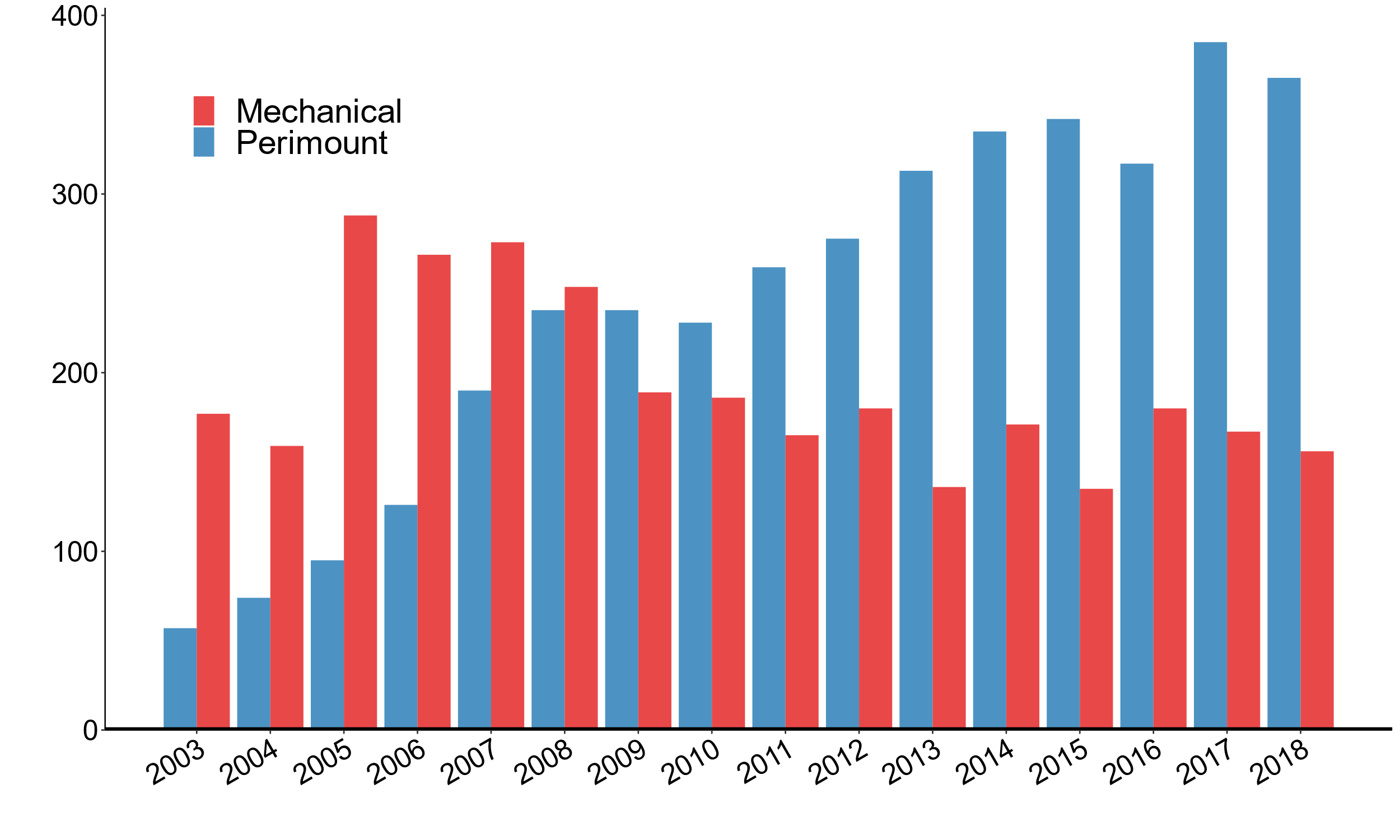
**

Supplemental Figure 2. Number of Perimount bioprosthetic valve subtype per year in patients aged 50–69 years in Sweden between 2003 to 2018.

**
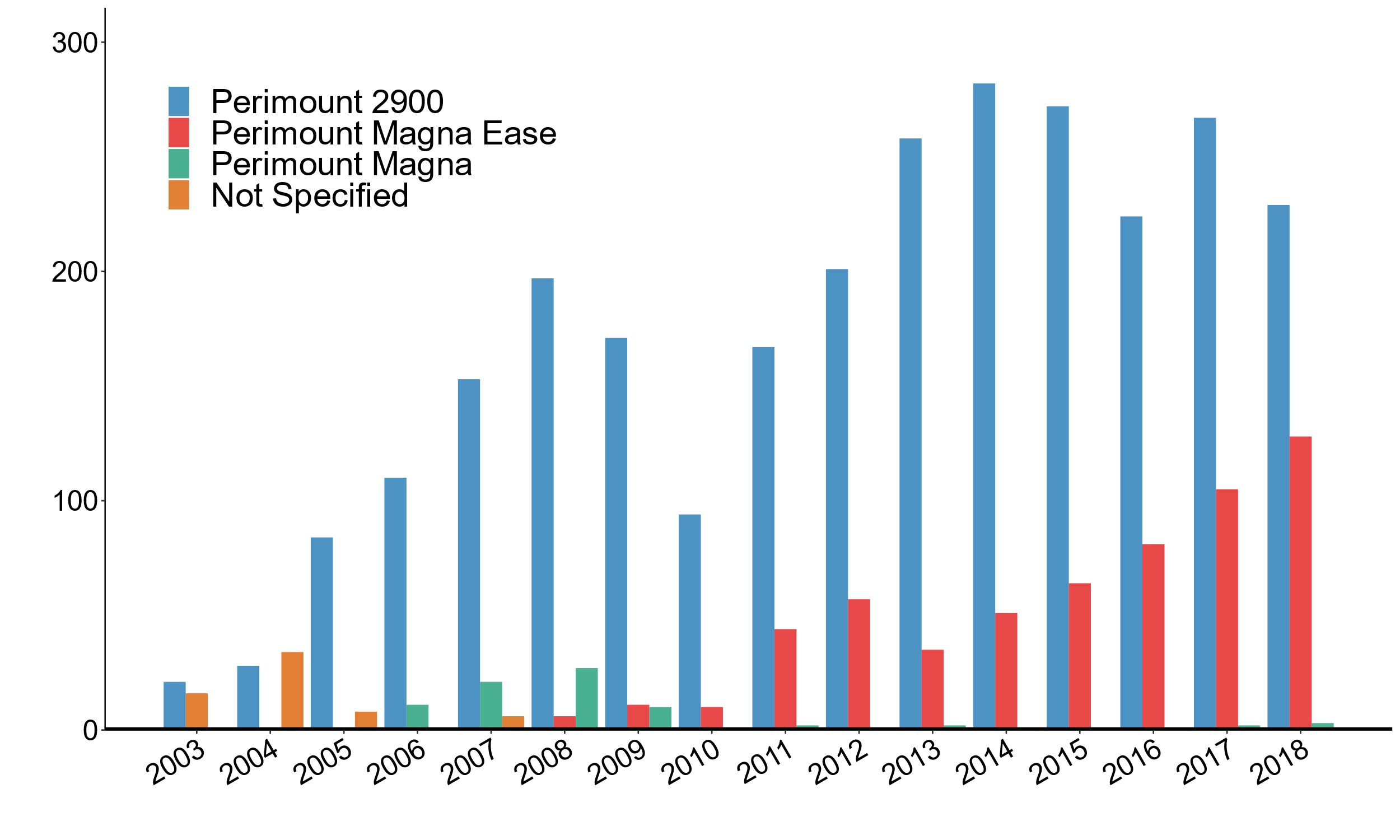
**

Supplemental Figure 3. Kaplan-Meier estimated survival in patients aged 50–69 years who underwent aortic valve replacement with a mechanical or Perimount valve in Sweden between 2003 to 2018.


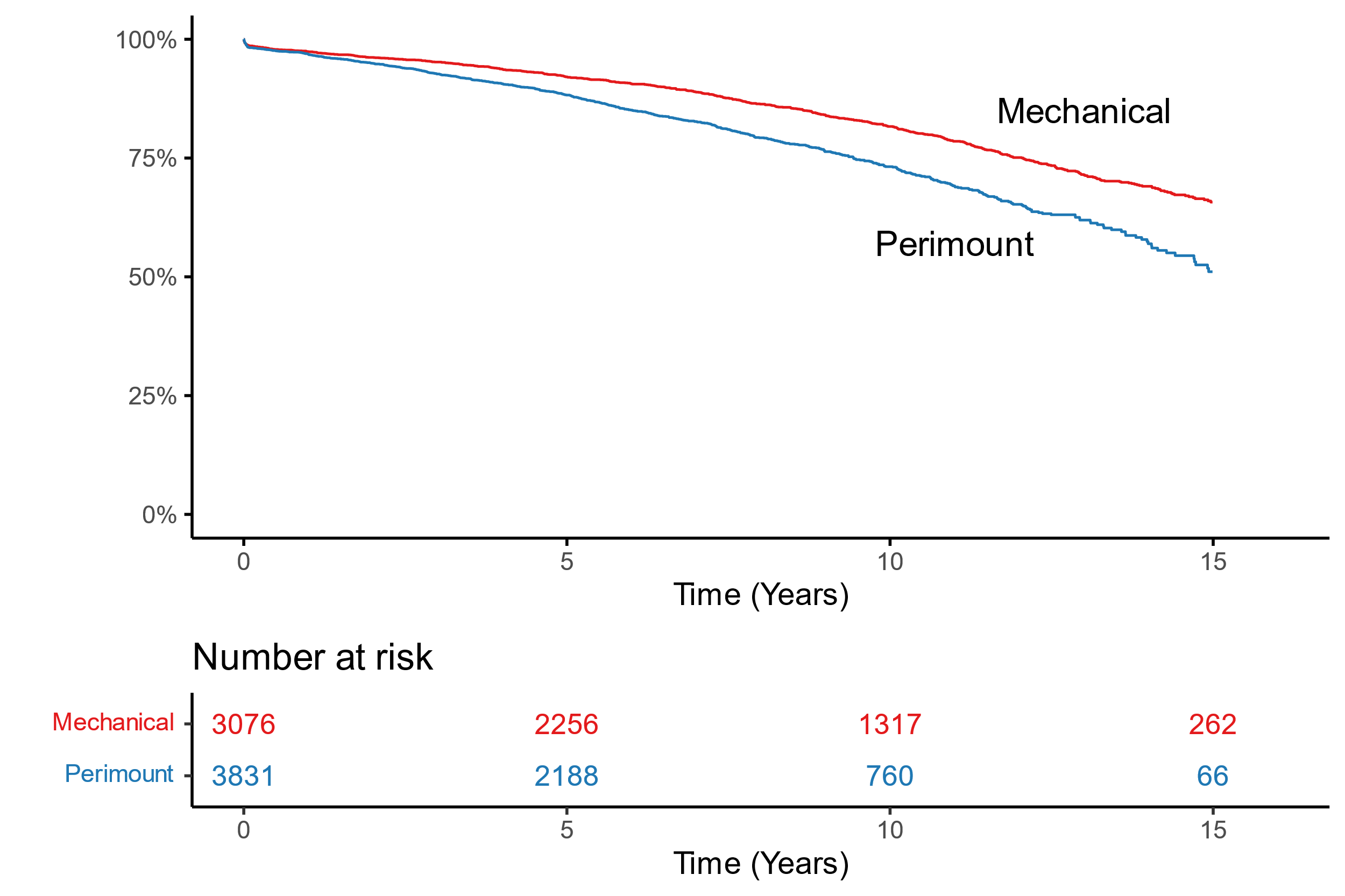


Supplemental Figure 4. Aalen-Johansen estimated crude cumulative incidence of complications in patients aged 50-69 who underwent aortic valve replacement with a mechanical or Perimount valve in Sweden between 2003 to 2018. Shaded areas represent 95% confidence intervals.

**
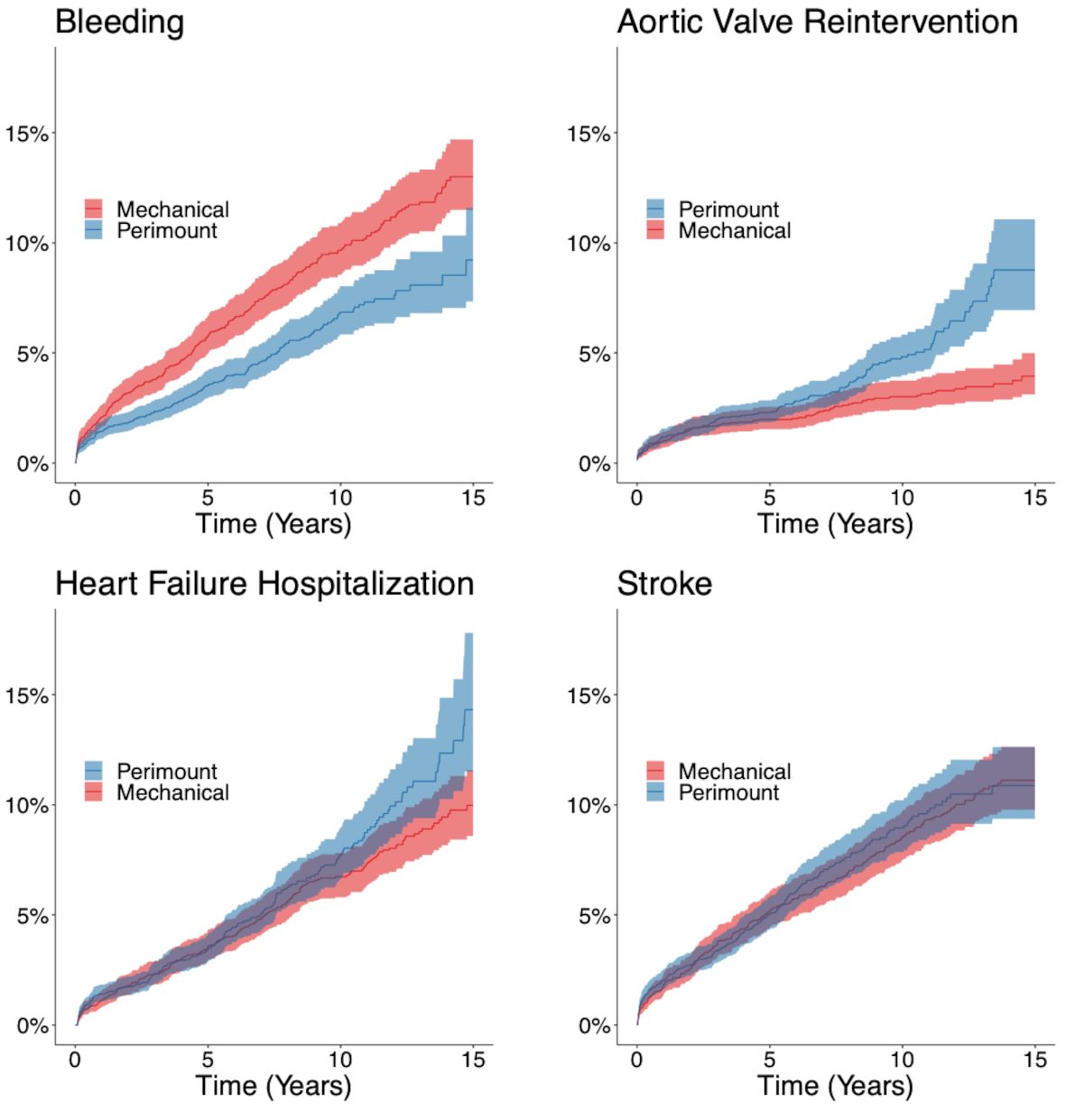
**

Supplemental Figure 5. Regression standardized complication rates in patients aged 50-59 who underwent aortic valve replacement with a mechanical or Perimount valve in Sweden between 2003 to 2018. Shaded areas represent 95% confidence intervals.


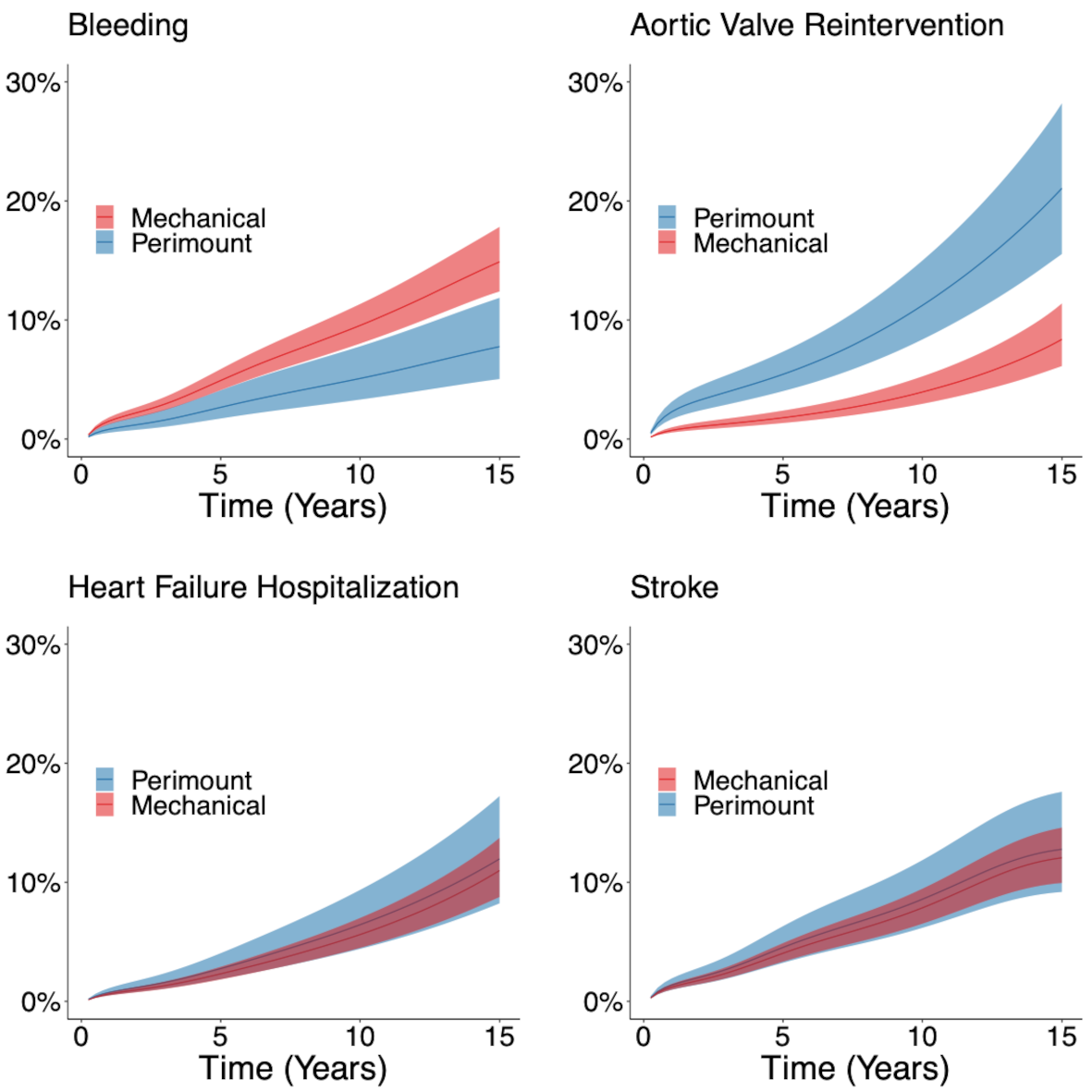


Supplemental Figure 6. Regression standardized complication rates in patients aged 60-69 who underwent aortic valve replacement with a mechanical or Perimount valve in Sweden between 2003 to 2018. Shaded areas represent 95% confidence intervals.

**
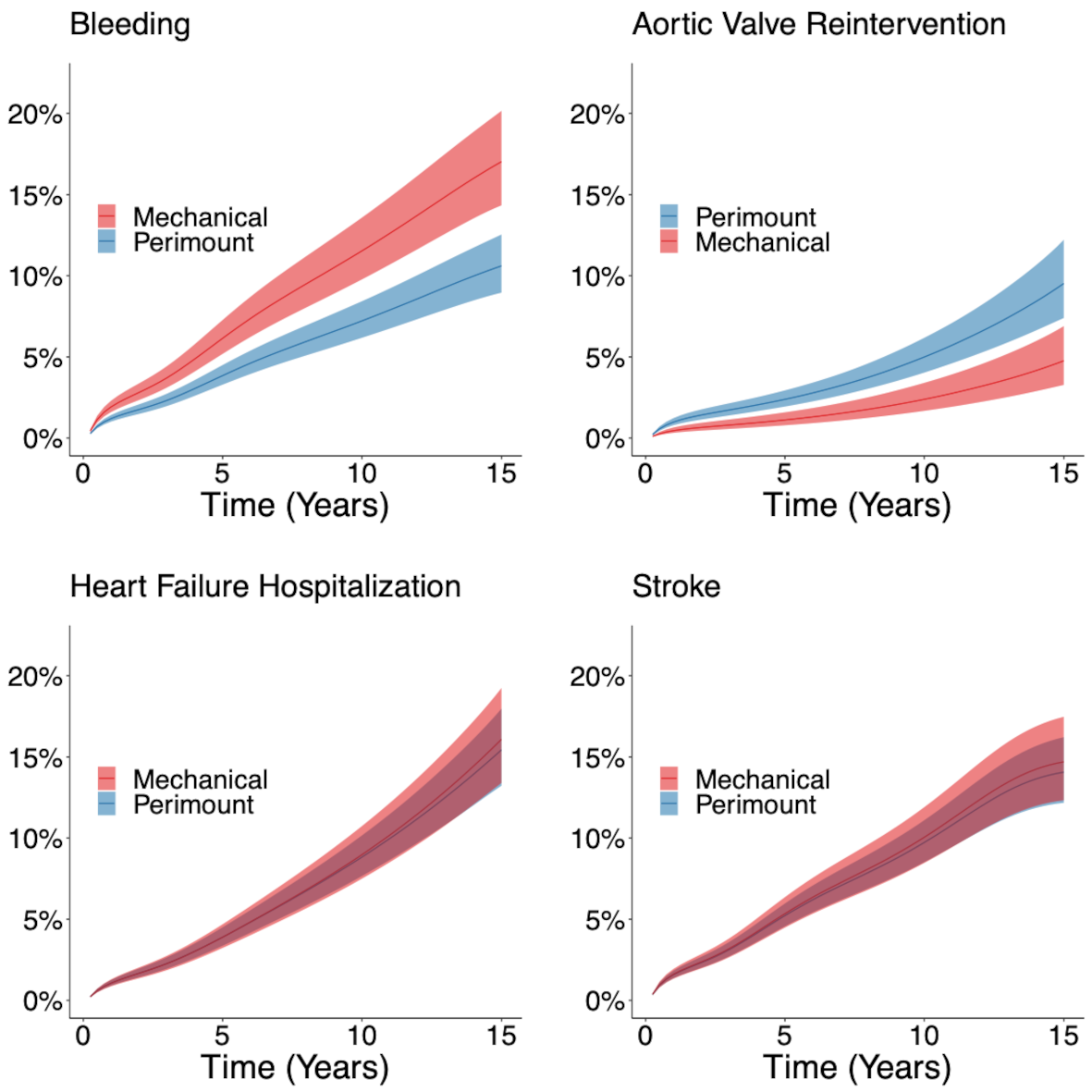
**
